# Clinical and neurocognitive outcome in symptomatic isovaleric acidemia

**DOI:** 10.1186/1750-1172-7-9

**Published:** 2012-01-25

**Authors:** Sarah C Grünert, Udo Wendel, Martin Lindner, Michael Leichsenring, K Otfried Schwab, Jerry Vockley, Willy Lehnert, Regina Ensenauer

**Affiliations:** 1 Center for Pediatrics and Adolescent Medicine, Albert-Ludwigs-Universität Freiburg, Germany; 2 Department of General Pediatrics, University Hospital Düsseldorf, Heinrich-Heine-Universität Düsseldorf, Germany; 3 Divison of Inborn Metabolic Diseases, University Children's Hospital, Universität Heidelberg, Germany; 4 Department of Pediatrics and Adolescent Medicine, University Medical Center Ulm, Germany; 5 Department of Pediatrics, University of Pittsburgh School of Medicine and Department of Human Genetics, University of Pittsburgh Graduate School of Public Health, Pittsburgh, Pennsylvania, USA; 6 Research Center, Dr. von Hauner Children's Hospital, Ludwig-Maximilians-Universität München, Germany

**Keywords:** isovaleric acidemia, symptomatic, neurocognitive outcome, mortality

## Abstract

**Background:**

Despite its first description over 40 years ago, knowledge of the clinical course of isovaleric acidemia (IVA), a disorder predisposing to severe acidotic episodes during catabolic stress, is still anecdotal. We aimed to investigate the phenotypic presentation and factors determining the neurological and neurocognitive outcomes of patients diagnosed with IVA following clinical manifestation.

**Methods:**

Retrospective data on 21 children and adults with symptomatic IVA diagnosed from 1976 to 1999 were analyzed for outcome determinants including age at diagnosis and number of catabolic episodes. Sixteen of 21 patients were evaluated cross-sectionally focusing on the neurological and neurocognitive status. Additionally, 155 cases of patients with IVA published in the international literature were reviewed and analyzed for outcome parameters including mortality.

**Results:**

57% of study patients (12/21) were diagnosed within the first weeks of life and 43% (9/21) in childhood. An acute metabolic attack was the main cause of diagnostic work-up. 44% of investigated study patients (7/16) showed mild motor dysfunction and only 19% (3/16) had cognitive deficits. No other organ complications were found. The patients' intelligence quotient was not related to the number of catabolic episodes but was inversely related to age at diagnosis. In published cases, mortality was high (33%) if associated with neonatal diagnosis, following manifestation at an average age of 7 days.

**Conclusions:**

Within the group of "classical" organic acidurias, IVA appears to be exceptional considering its milder neuropathologic implications. The potential to avoid neonatal mortality and to improve neurologic and cognitive outcome under early treatment reinforces IVA to be qualified for newborn screening.

## Background

Isovaleric acidemia (IVA) is known as one of the "classical" organic acidemias/acidurias. It is caused by a genetic deficiency of isovaleryl-CoA dehydrogenase (IVD) catalyzing the third step in leucine catabolism. The enzyme defect results in the accumulation of derivatives of isovaleryl-CoA including free isovaleric acid, 3-hydroxyisovaleric acid, isovaleryl (C5)-carnitine, and isovalerylglycine (IVG) which partly may exert neurotoxicity.

The clinical presentation of IVA appears to be highly variable ranging from severely affected to asymptomatic subjects [1]. It may present either in the neonatal period as an acute episode of fulminant metabolic acidosis which may lead to coma and death ("acute neonatal form") or later as a "chronic intermittent form" associated with developmental delay, with or without recurrent acidotic episodes during catabolic stress. Proposed strategies for long-term treatment of IVA comprise protein or leucine restriction to reduce the production of toxic metabolites from leucine degradation, and carnitine and/or glycine to enhance the conversion of potentially neurotoxic free isovaleric acid into non-toxic carnitine and glycine conjugates which are readily excreted in the urine [2,3].

Recently, the identification of a novel mild and potentially asymptomatic form of IVA and its association with a common missense mutation, c.932C>T (p.A282V), was reported [1]. This type of IVA is frequent in subjects identified through newborn screening (NBS) and associated with less pronounced metabolite elevations than in clinically detected patients. This goes along with the finding of a higher incidence of IVA in the NBS population than in clinically detected cases [4].

Although the detection of various metabolic disorders including IVA by NBS using tandem mass spectrometry is technically feasible [5], policies for the inclusion of disorders in NBS programs world-wide are diverse and remain a matter of debate. With respect to IVA, there is an entire lack of larger-scale studies investigating the long-term outcome in clinically diagnosed patients. A better understanding of the clinical course of IVA is therefore required in order to allow for 1) an evidence-based decision-making on the inclusion of IVA in NBS programs and 2) a prospective assessment of patient outcome in countries in which IVA has already been incorporated into the NBS disease panel. This study provides data on the largest series of patients with IVA reported to date highlighting the clinical course of the disease and the neurological and cognitive outcome.

## Methods

### Study design and patients

Twenty-one patients with IVA from 21 families living in Germany were enrolled in a cross-sectional study (Table 1). All were diagnosed due to clinical presentation between 1976 and 1999, prior to the inclusion of IVA as a target disorder in German NBS programs. In all cases, IVA was confirmed by demonstrating IVA-specific metabolites in plasma and/or urine.

**Table 1:** Characteristics of study patients with symptomatic isovaleric acidemia.

ID	Age at study (y)	Sex	Ethnicity	Age at diagnosis	**Frequency of catabolic episodes**^b^			**Medical long-term treatment**^c^			**Leucine-free amino acid supplementation**^d^
						
					SE	MO	IM	C	C+G	none	
1	2.2	M	DE	1 w	0	1	0		X		-

2	3.8	M	DE	1.6 y	0	1	1		X		X

3	5.3	F	TR	1 w	0	5	0		X		-

4	6.1	F	DE	3.7 y	0	2	2	X			-

5	6.5	F	DE	1 w	1	2	0	X			X

6	7.1	F	DE	1 w	1	5	3		X		X

7	7.5	M	DE	1 w	1	0	1		X		-

8	8.4	M	TR	6.3 y	1	1	2		X		-

9	10.7	F	TR	1 w	1	5	2	X			X

10	10.9	F	TR	1 w	2	8	3	X			X

11^a^	11.5	F	GR	4.5 y	0	1	0		X		-

12	11.6	M	DE	2 w	1	2	0		X		Until age 5 y

13	14.2	M	DE	2.4 y	2	0	0		X		-

14	15.4	M	DE	5 w	0	2	2	X			Until age 15 y

15^a ^	16.5	F	TR	2.4 y	1	1	0	X			-

16	19.0	F	IT	1 w	1	5	0	X			During infancy

17^a ^	20.0	M	TR	5.3 y	0	5	0	X			-

18^a ^	20.0	F	TR	3.7 y	0	1	4	NA	NA	NA	-

19	24.4	M	DE	1 w	0	4	2			X	During infancy

20	25.3	F	GR	6.5 y	0	5	2			X	-

21^a ^	Deceased	M	DE	2 w	1	0	0	-	-	-	-

Information on the clinical presentation including age at diagnosis, number of catabolic episodes, and treatment modalities is detailed.

C = L-carnitine, F = female, G = L-glycine, DE = German, GR = Greek, IT = Italian, ID = identification number, IM = impending catabolic episode, M = male, MO = moderate catabolic episode, NA = not available, SE = severe catabolic episode, TR = Turkish, w = week(s), y = years

^a ^Patient not participating in study step II; information was available from questionnaire and/or medical records only.

^b ^Total number of catabolic episodes (as defined in the Methods section), both prior to and following the diagnosis.

^c ^L-carnitine intake ranged from 10 to 144 mg/kg body weight per day (median 100 mg/kg × d); L-glycine was administered in doses between 53 and 200 mg/kg body weight per day (median 112 mg/kg × d).

^d ^All patients were on a protein-restricted diet.

**Figure 1:**
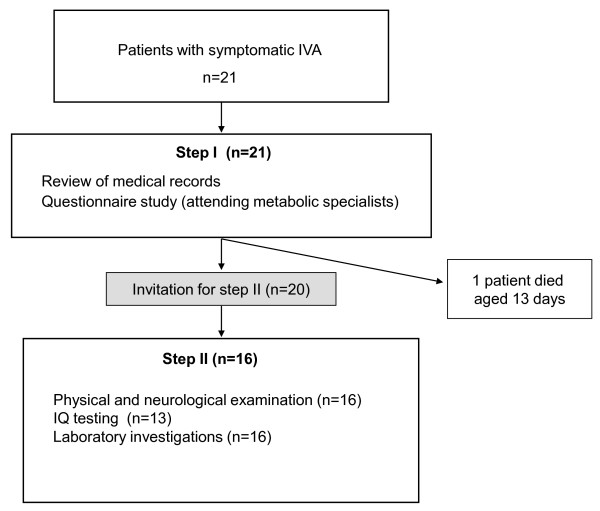
**Study design**. Sixteen of a total of 21 study patients with symptomatic isovaleric acidemia (IVA) were evaluated not only by use of a questionnaire and/or medical records (step I) but also by cross-sectional clinical, biochemical, and neurocognitive assessments (step II). IQ = intelligence quotient.

The study was conducted in two steps (Figure 1). In a first step, clinical information was collected for each patient by review of the patients' medical records and use of a questionnaire which was sent to the patients' attending metabolic specialists, specifically addressing the patients' age at diagnosis, catabolic episodes (frequency, severity, and triggers), clinical findings, sociodemographic status, psychomotor and cognitive development as well as biochemical and treatment data. For one patient the relevant information was obtained only *post mortem*. Next, 20 patients were invited to take part in the second step of the study comprising physical examination, cognitive testing, and laboratory investigations. Sixteen patients agreed to participate. The participants were examined during a one-day study visit at the University Children's Hospital in Freiburg, Germany (n = 12), or if this was impossible, at the pediatric metabolic department in charge (n = 4), within a study period of 10 months. Informed consent was obtained from the patients and/or patients' parents. The study was approved by the Institutional Review Board of the University Freiburg, Germany.

### Systematic literature review

We performed a literature review by searching PubMed using the terms "isovaleric acidemia", "isovaleric aciduria", "organic acidemia", and "organic aciduria" in order to obtain comprehensive information on the clinical course of IVA from a larger number of patients. 155 cases of clinically diagnosed IVA published between January 1967 and June 2011 were identified and reviewed, specifically focusing on the patients' age of disease onset, mortality, and neurocognitive outcome (Additional file 1, Table S1).

### Definition of the terms "time of diagnosis" and "catabolic episode"

The term "early diagnosis" refers to a diagnosis made within the first 5 weeks of age, whereas the term "late diagnosis" refers to a diagnosis made thereafter (Table 1). Patients with IVA are prone to recurrent episodes of metabolic acidosis following catabolic stress e.g. during intercurrent illness, but sometimes without any overt cause. In between, they may be without pathological findings. To estimate the impact of catabolic episodes on the patients' outcome, three degrees of episode severity were defined. The term "severe catabolic episode" refers to an acute attack of metabolic acidosis associated with apathy or coma and neurological signs (e.g. seizures) requiring intensive supportive treatment. "Moderate catabolic episode" refers to an acute attack of illness with at least one clinical (e.g. recurrent emesis, lethargy) and/or biochemical (e.g. metabolic acidosis) sign not requiring intensive care treatment. "Impending catabolic episode" is defined as acute illness not distinguishable from a common infection without metabolic acidosis (Table 1).

### Clinical examination

All step II participants underwent a careful physical and neurological examination including anthropometric assessments. Neurological examination comprised assessment of gait and station, deep tendon reflexes, motor coordination, gross motor function, and cranial nerve function as well as, in patients older than 5 years, diadochokinesis of the upper and lower limbs.

### Evaluation of psychomotor development and cognitive performance

In step II participants aged 1 to 3.8 years, psychomotor development was evaluated using the Denver Developmental Screening Test (DDST) (Table 2). To determine the intelligence quotient (IQ) in older step II participants, the age-appropriate German versions of the following IQ tests were applied: in children aged 4 to 12 years the Kaufman Assessment Battery for Children (K-ABC) and in one case the Kramer IQ test (Kramer); in the remaining adolescent and adult patients the Wechsler Intelligence Scale for Children Version III (HAWIK-III) and the Wechsler Adult Intelligence Scale-Revised (HAWIE-R), respectively. In one step II participant with severe retardation in whom testing was not possible and four patients not participating in step II, information on psychomotor development and/or IQ was obtained from the questionnaire and medical records (Table 2).

**Table 2:** Psychomotor development and cognitive performance of study patients with symptomatic isovaleric acidemia.

ID	Age at evaluation (y)	**Psychomotor development**^b^	IQ score	**IQ testing tool**^d^
1	2.2	Normal	NA	DDST

2	3.8	Mild retardation	NA	DDST

3	5.3	Normal	108	K-ABC

4	6.1	Normal	106	K-ABC

5	6.5	Normal	116	K-ABC

6	7.1	Normal	108	Kramer

7	7.5	Normal	99	K-ABC

8	8.4	Learning disability	90	K-ABC

9	10.7	Learning disability	99	K-ABC

10	10.9	Mild retardation	67	K-ABC

11^a^	11.5	Normal	NA	NA

12	11.6	Normal	118	K-ABC

13	14.2	Learning disability	85	HAWIK-III

14	15.4	Normal	116	HAWIE-R

15^a^	16.5	Learning disability	93/94^c^, tested at age 8.5 y	CPM/CMMS

16	19	Normal	119	HAWIE-R

17^a^	20	Normal	101/91^c^, tested at age 11.6 y	SON/SPM

18^a^	20	Normal, speech retardation at age 3.7 y	NA	NA

19	24.4	Normal	122	HAWIE-R

20	25.3	Severe retardation	51/45^c^, tested at age 6.8 y	HAWIK/Kramer

Information is provided for a total number of 20 patients (one death at 13 days of life).

ID = identification number, IQ = intelligence quotient, NA = not available, y = years

^a ^Patient not participating in study step II.

^b ^According to medical records/questionnaire and, in case of participation in study step II, clinical investigation.

^c ^IQ testing not performed at study step II; information on IQ available from medical records/questionnaire only.

^d ^CMMS = Columbia Mental Maturity Scale (Mean ± SD: 100 ± 16), CPM = Raven's Coloured Progressive Matrices (Mean ± SD: 100 ± 15), DDST = Denver Developmental Screening test, HAWIE-R = Wechsler Adult Intelligence Scale-Revised (Mean ± SD: 100 ± 15), HAWIK = Wechsler Intelligence Scale for Children (Mean ± SD: 100 ± 15), HAWIK-III = Wechsler Intelligence Scale for Children Version III (Mean ± SD: 100 ± 15), K-ABC = Kaufman Assessment Battery for Children (Mean ± SD: 100 ± 15), Kramer = Kramer IQ test (Mean ± SD: 100 ± 15), NA = not available, SPM = Raven's Standard Progressive Matrices (Mean ± SD: 100 ± 15), SON = Snijders-Oomen Nichtverbaler Intelligenztest-R 5^1^/_2_-17 (Mean ± SD: 114 ± 14).

### Laboratory investigations

In all 16 step II participants, routine laboratory tests including vitamins and minerals as well as plasma amino acids were performed in order to screen for various organ involvements and potential nutritional deficits, as they were on a protein-restricted diet (Table 1; Additional file 2, Table S2). In addition, the following disease-specific biochemical investigations were carried out: quantification of the IVD activity in the patients' Epstein-Barr virus-transformed lymphocytes using the anaerobic electron-transfer flavoprotein reduction assay [6], measurement of the C5-carnitine accumulation in the patients' lymphocyte culture medium as described previously [1], and quantification of IVG excreted in urine (Additional file 2, Table S2).

### Statistical analysis

Statistical analyses were performed using SPSS 16.0 (2007, IBM, Chicago, Illinois). For the determination of the correlation of patients' IQ scores with the number of catabolic episodes, linear correlation analyses were performed using the Kendall tau coefficient. Correlation of patients' IQ scores with age at diagnosis was determined using the Pearson coefficient, and the relative impact of these outcome determinants on patients' IQ scores was assessed by linear regression analyses. The Mann-Whitney U test was used to assess differences in age at initial manifestation between deceased and surviving patients within the early diagnosis group of literature patients. *P *< 0.05 was considered statistically significant.

## Results

### *Study patients*

### Characteristics

The age of 20 living patients entering step I of the study ranged from 2 to 25 years (median 11.2 years). One patient died at the age of 13 days (Table 1, patient ID 21). Sixteen (76%) patients were in the pediatric and adolescent age group (< 18 years), five patients were in the adult age group (19 to 25 years). The male to female ratio was 0.9. Eleven patients were natives; the remainder were migrants of Turkish, Greek, and Italian origin. The parental consanguinity rate was 48% (10/21) overall, with a higher rate (80%, 8/10) in migrant families. Three patients (14%, 3/21) had living siblings also affected with IVA. In three families (14%, 3/21), unexplained death of a preceding sibling had occurred within the first 3 months of life. In all 21 study patients, pregnancy, birth, and early postnatal period were uneventful and birth weight was within normal limits.

### Biochemical phenotype

Both the IVD activity in the patients' transformed lymphocytes measured below the detection limit and the greatly increased concentration of C5-carnitine in the culture medium of the lymphocytes (0.31 to 1.3 μmol/g protein; < 0.01 in healthy controls, [1]) confirmed the diagnosis of IVA for all patients. The biochemically severe phenotype was also reflected by the high urinary IVG excretion, ranging from 685 to 2.101 mmol/mol creatinine (median: 1.256 mmol/mol creatinine; < 10 in healthy controls) in patients not receiving glycine supplementation (n = 10, Table 1).

### Age at diagnosis

Twelve patients (12/21, 57%) were diagnosed within the first 5 weeks of life, the majority of whom within the first week (9/12, 75%) (Table 1). Symptoms ranged from poor feeding and emesis to severe acidosis accompanied by neurological signs and apathy. In one patient, the initial attack of severe metabolic acidosis was lethal. In the remaining nine patients (9/21, 43%), the diagnosis was made after the first year of life due to an acute attack in all but two who had diagnostic work-up for developmental delay. However, nearly all of the late diagnosed patients in whom information was available (7/8; 88%) also had experienced episodic symptoms during the neonatal period including vomiting and feeding difficulties requiring treatment.

### Catabolic episodes

**Figure 2:**
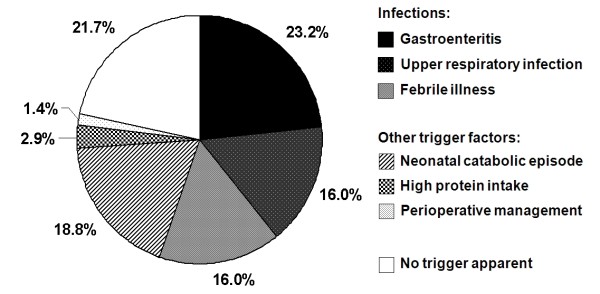
**Triggering factors of catabolic episodes in symptomatic isovaleric acidemia (IVA)**. Moderate and severe catabolic episodes (n = 69) in 21 study patients with symptomatic IVA were analyzed as documented in the medical records and/or questionnaires. No specific focus of infection was evident in febrile illnesses.

**Figure 3:**
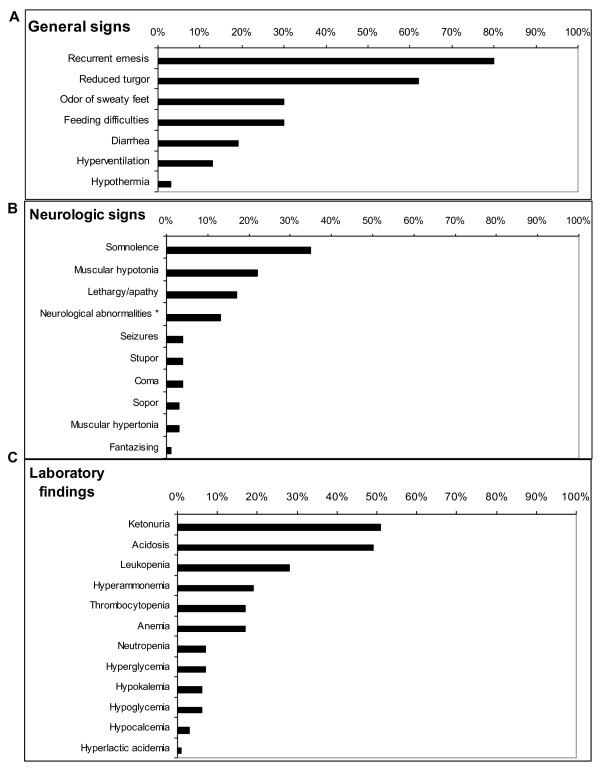
**Clinical signs and laboratory findings at catabolic episodes in symptomatic isovaleric acidemia (IVA)**. Prevalence of clinical signs and laboratory findings during moderate and severe catabolic episodes (n = 69) of 21 study patients with symptomatic IVA were analyzed as documented in the medical records and/or questionnaires. A) General clinical signs; B) neurological findings; C) clinical chemical and hematologic abnormalities measured in blood and urine at the time of episodes. * Neurologic abnormalities included hyperexcitability, dyscoordination, decreased tendon reflexes.

Overall, 69 documented moderate and severe catabolic episodes were reported for the 21 study patients (Table 1), 41 (59%) thereof occurred in 13 study patients after the diagnosis of IVA was made and therapy was initiated. In most patients, the frequency of catabolic episodes was highest during early infancy, with approximately one-fifth occurrence in the neonatal period (Figure 2), and decreased with age. No apparent catabolic episode was observed after 9 years of age. Most common signs during episodes were recurrent vomiting and somnolence (Figure 3). Gastroenteritis was the most common trigger, while protein excess or surgery did not play a major role as precipitating factors (Figure 2). In approximately one-fifth of catabolic episodes, no trigger was apparent.

### Clinical outcome

For a total of six study patients, a concomitant disorder was specified in the medical records/questionnaires. One patient each suffered from bilateral cochlear deafness, hypothyroidism, a small ventricular septum defect, and pancreatitis. Two patients had undergone pylorotomy in infancy due to a diagnosis of hypertrophic pyloric stenosis. The physical examination was normal in the 16 patients examined during step II of the study; specifically, no clinical signs of organ involvement were observed. In all patients, growth was within normal limits. In nine patients, the neurologic examination was entirely normal, whereas seven patients showed signs of mild dyscoordination or mild motor dysfunction. Laboratory findings (Additional file 2, Table S2) were indicative neither of specific organ involvement nor relevant nutritional deficiencies.

### Psychomotor development and neurocognitive outcome

Data on psychomotor development as obtained for 20 patients from the medical records and questionnaires (step I of the study) were predominantly normal. In two patients mild and in one severe psychomotor retardation was noted (Table 2, patient IDs 2, 10, 20). An early speech development disorder was recorded in one patient (ID 18).

The cognitive performance in terms of IQ was obtained in 13 of the 16 step II participants aged 5 to 24 years, whereas younger children were evaluated using DDST (Table 2). Besides cognitive impairment in the three retarded patients mentioned above (3/16, 19%), we identified one patient with a known diagnosis of a learning disability to be in the low-normal IQ range (HAWIK-III 85). However, two other step II participants with learning disabilities were tested normal, close to the average IQ (Table 2).

Analyzing the impact of the time at diagnosis and the number of catabolic episodes on the IQ, it turned out that the IQ of the 13 step II participants was not related to the number of documented moderate/severe catabolic episodes (*r *= 0.12, beta = -2.15 [-5.67, 1.38], *p *= 0.26). However, there was a significant inverse relationship between the IQ score and age at diagnosis (in days) in patients with not more than one severe catabolic episode (*r *= -0.63, beta = -0.008 [-0.001, -0.015], *p *< 0.05, n = 11).

**Figure 4:**
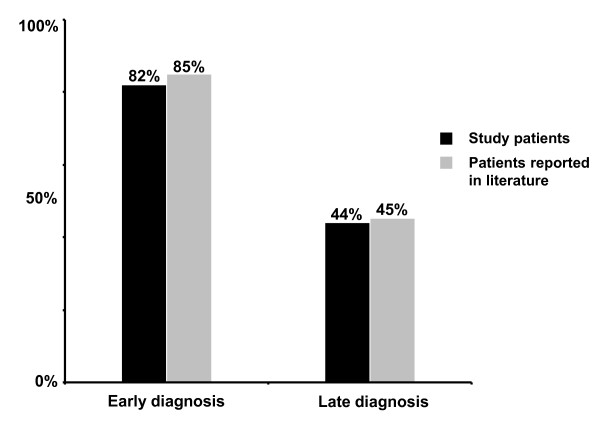
**Proportion of patients with an unremarkable neurocognitive outcome in symptomatic isovaleric acidemia**. The percentage of patients with normal cognitive abilities (intelligence quotient [IQ] scores) including academic performance (absence of learning disabilities) is presented for the group of study patients (n = 20). In the group of patients reported in the literature (n = 108), neurocognitive outcome was defined as normal if reported as "normal" or "excellent" and/or normal IQ scores were available. Patients were subdivided into an early diagnosis group (defined as a diagnosis made within the first 5 weeks of life) and a late diagnosis group (defined as a diagnosis made thereafter). Of 20 study patients, 11 were diagnosed early and nine were diagnosed late (median 3.7 years; range 1.6-6.5 years). Of 108 patients in whom information on neurocognitive outcome was available in the literature, 46 were diagnosed early and 62 were diagnosed late.

When subgrouped according to age at diagnosis, normal cognitive abilities (IQ scores) including academic performance (absence of a learning disability) were evident in 82% (9/11) of the early diagnosed study patients compared to only 44% (4/9) of late diagnosed study patients (Figure 4). Furthermore, learning disabilities were reported in three of nine (33%) late diagnosed study patients (IDs 8, 13, 15), while only one was affected in the early diagnosis group (9%; 1/11, ID 9) (Tables 1 and 2). The only patient who was severely mentally retarded was diagnosed late at the age of 6.5 years (ID 20).

### *Patients of the literature review*

Data from 155 patients with IVA were extracted from the international literature (Additional file 1, Table S1). Eighty-one patients were diagnosed early within the first 5 weeks of life, whereas 74 patients were diagnosed late, seven within the first year and most of the remainder between 1 and 6 years of age.

### Mortality

**Figure 5:**
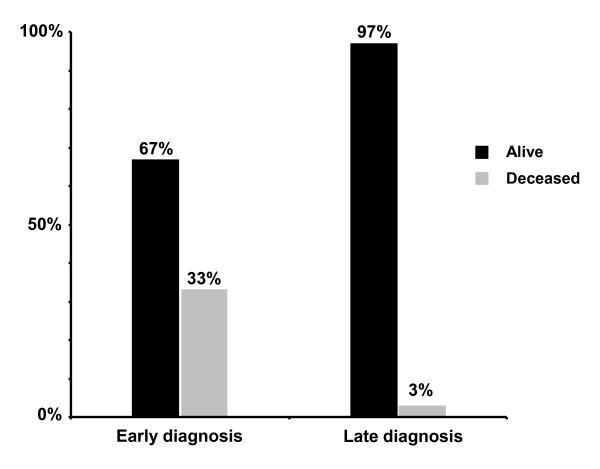
**Survival data of 155 patients with symptomatic isovaleric acidemia reported in the literature**. Early diagnosis was defined as a diagnosis made within the first 5 weeks of life (n = 81), late diagnosis was defined as a diagnosis made thereafter (n = 74).

Mortality in the early diagnosis group was 33% during the initial episode of severe metabolic acidosis (Figure 5; Additional file 1, Table S1). In the early diagnosis group, the mean age at onset of symptoms as reported for 55 of 81 cases, was 6.8 ± 5.4 days (mean ± SD; median 6 days). Amongst them, 20 patients who died during the initial catabolic episode had a significantly earlier onset of symptoms (mean ± SD: 4.6 ± 2.9 days; median 3.5 days) than 35 patients who survived this initial catabolic episode (mean ± SD: 8.1 ± 6.1 days; median 7 days) (*p *< 0.05). In patients diagnosed late, the mortality rate was only 3% (Figure 5).

### Neurocognitive outcome

Data on the neurocognitive outcome were reported for 108 patients (Additional file 1, Table S1). Of patients with an early diagnosis and treatment, 85% (39/46) had a normal cognitive outcome, whereas only 45% (28/62) of patients with a late diagnosis had a normal cognitive outcome (Figure 4).

## Discussion

Although IVA is known for a number of decades as a potentially life-threatening metabolic disease and included in NBS programs, information has remained sparse as to the clinical course and outcome. This large set of outcome data in patients with symptomatic IVA provides a basis to fill this gap. The main findings are that 1) the mortality rate is high in association with early neonatal presentation; 2) the neurocognitive outcome in surviving patients with neonatal manifestation and early start of treatment is more favourable than in patients with a late diagnosis; and 3) age at diagnosis but not the number of catabolic episodes has an impact on the neurocognitive outcome.

### Mortality associated with neonatal manifestation is high but survivors benefit from early diagnosis and treatment

Analyzing the course of 155 cases with clinically evident IVA published over a period of more than 40 years, we found that the mortality in neonates during their initial catabolic episode was high, with 30% much higher than during catabolic episodes in patients who were diagnosed later during infancy or childhood. In patients presenting acutely in the neonatal period, the mean age at onset of the first potentially life-threatening catabolic episode was 6.8 days and was even earlier (4.6 days) in patients who died during the initial catabolic episode. A way out of this high mortality rate in the past can be expected through the addition of IVA as a target disease to NBS programs, which recently took place in a number of countries. At least in the German and various European NBS programs, blood sampling of the newborn is to be performed between 36 and 72 hours of life, and the communication of a positive screening result can thus be expected to take place between day 4 and 7 of life. Hence, for many patients with an acute neonatal manifestation of IVA, the NBS result arrives in time to prevent severe clinical manifestation and exert the proper treatment, thus bearing the potential of reducing early mortality.

Looking at the psychomotor development and neurocognitive outcome in later life, study patients who survived an acute neonatal manifestation appear to benefit from early diagnosis and start of therapeutic measures including a protein-restricted diet and carnitine/glycine supplementation. These patients showed later a better neurocognitive outcome than patients who were not diagnosed before childhood. This was in accordance with the results of our literature review based on a large series of patients with IVA (Figure 4).

When the overall neurocognitive outcome of the disorder is considered, it appears less devastating compared to other "classical" organic acidemias/acidurias such as methylmalonic aciduria (MMA) and propionic acidemia (PA) [7,8]. In our own IVA study population, neurocognitive development was favourable in more than 80% of patients (13/16) as assessed by developmental/IQ testing. However, despite normal IQ outcome, minor disabilities including reported learning disability and earlier speech retardation were evident in a significant proportion (25%) of the study patients (5/20). Exclusion of these cases led to a percentage of 60% of patients (12/20) with an entirely normal neurocognitive outcome/psychomotor development and unremarkable academic performance. This is in agreement with data obtained from our literature review: 62% (67/108) of patients with IVA, either diagnosed early or late, were reported to have a normal neurocognitive outcome while the remainder of patients was impaired (Additional file 1, Table S1). Severe mental retardation was reported in only six (4%) of 155 patients, similar to our study population (5%).

No significant long-term complications beyond the central nervous system (CNS) involvement - mild motor dysfunction and cognitive deficits - were evident in our study patient population. The majority of patients showed a normal physical development. Similar observations have been reported by Martin-Hernandez et al [9] who studied four adult IVA patients. All of them had normal growth and none showed visceral complications. Thus, in contrast to MMA or PA in which long-term complications like failure to thrive [10], renal failure [7], or cardiac complications [11] are common, IVA does not seem to be a multisystemic disorder. Besides its beneficial neurocognitive outcome, the high potential of a normal physical development without restrictive long-term complications in affected patients additionally emphasizes the value of early, pre-symptomatic diagnosis by means of NBS.

### The neurocognitive outcome is not related to the number of catabolic episodes

Among several factors suggested to influence the long-term outcome of patients with IVA including genotype [1], residual IVD activity, and quality of therapy, the number and severity of catabolic episodes might determine the neurocognitive outcome. However, in the present study no significant relationship between the number of moderate and severe catabolic episodes the patients had experienced and their IQ at study was found. Some patients had normal IQs despite severe catabolic episodes with coma while others with an apparently milder clinical course showed cognitive impairment. Similar observations have been made in patients reported in the literature [9,12-14].

The underlying pathophysiologic mechanisms leading to cerebral damage are still not fully elucidated. Based on studies in rats it can be presumed that metabolites accumulating in IVA induce oxidative stress in the brain cortex and that oxidative damage may be at least in part involved in the neuropathology of IVA [15]. Additionally, it was recently shown that particularly free isovaleric acid reduces Na+, K+-ATPase activity in synaptic membranes from cerebral cortex of young rats, possibly via mechanisms involving lipid peroxidation [16]. It might be hypothesized that every severe catabolic episode causes cerebral damage to some extent by means of the accumulating free isovaleric acid. However, considering the lack of association between the number of catabolic episodes and cognitive performance (IQ) and the finding that early diagnosis and treatment have a positive impact on the neurocognitive outcome, chronic rather than acute damage might be the predominant factor determining the extent of neuropathology in patients with IVA. A possible explanation might be that in patients on treatment, most of the persistently accumulating and potentially neurotoxic free isovaleric acid is converted into non-toxic carnitine and glycine conjugates. This implies that patients with the later manifesting form of IVA will also benefit from early diagnosis by NBS.

Nevertheless, patients with a clinically relevant form of IVA are constantly at risk of severe, potentially life-threatening acidotic decompensations during catabolic stress. As in other "classical" organic acidemias/acidurias the frequency of metabolic attacks is highest during early infancy and subsequently decreases with age [17]. In our patient population no catabolic episodes were observed after the age of 9 years. This is in accordance with a recent report of four adult IVA patients by Martin-Hernandez et al [9], who also have remained metabolically stable since adolescence. This might be partly explained by the reduced number of infections, a relevant triggering factor as shown here, with age [17]. Additionally, it has been discussed that alternative metabolic pathways leading to detoxification of accumulating metabolites in IVA can be better utilized with age [18].

However, sparse information on adolescent and adult patients in the literature indicates that even patients who have been metabolically stable for more than one decade are still prone to metabolic decompensation in episodes of catabolic stress [19-21]. Towards adolescence and adulthood, trigger factors may change and comprise general anesthesia, excessive sports and physical exertion, low-calorie diets and fasting, pregnancy, and alcohol potentially resulting in a catabolic state with increased endogenous protein turnover and the risk of potentially life-threatening crises. Thus, besides continuation of treatment, awareness of the disease risks is a life-long essential in patients with symptomatic IVA.

## Conclusion

Within the entity of "classical" organic acidurias which typically result in significant CNS damage, IVA appears to be exceptional considering its milder neuropathologic implications, possibly entirely preventable by treatment. The potential to avoid early mortality and to improve neurocognitive outcome by early diagnosis and treatment encourages pre-symptomatic diagnosis and reinforces IVA to be qualified for NBS.

## Competing interests

The authors declare that they have no competing interests.

## Authors' contributions

SCG collected data and drafted the manuscript; UW interpreted data, drafted/revised the manuscript; ML, MLe, KOS, WL were involved in the collection of data and reviewed/edited the manuscript; JV analyzed data, contributed to discussion, and reviewed/edited the manuscript; RE designed study, was involved in the collection of data, analyzed and interpreted data, drafted/revised the manuscript. All authors read and approved the final manuscript.

## Supplementary Material

Additional file 1 **Table S1: Literature review of 155 reported patients with symptomatic isovaleric acidemia; Supplementary References**. Click here for file

Additional file 2 **Table S2: Clinical chemical and biochemical parameters analyzed in step II participants with symptomatic isovaleric acidemia**. Click here for file
