# Supplementary material for: Clinical and neurocognitive outcome in symptomatic isovaleric acidemia
Source: Orphanet J Rare Dis. 2012 Jan 25;7:9. doi: 10.1186/1750-1172-7-9 (PMC3292949; doi:10.1186/1750-1172-7-9)
Supplement: Additional file 1 — Table S1: Literature review of 155 reported patients with symptomatic isovaleric acidemia; Supplementary References. [file 1750-1172-7-9-S1.DOC]

**Table S1:** **Literature review of 155 reported patients with symptomatic isovaleric acidemia.** “Early diagnosis” was defined as a diagnosis made within the first 5 weeks of life, “late diagnosis” was defined as a diagnosis made thereafter. The neurocognitive outcome is presented as stated in the original publications. Neurocognitive outcome was defined as “normal” if reported as “normal" or "excellent” or normal intelligence quotient (IQ)/ developmental quotient (DQ) scores were provided. It was defined as “impaired” if reported as “delayed” or “retarded” (various degrees) or if IQ/DQ scores below the normal range were recorded. If no details on the neurocognitive outcome, age at onset, or catabolic episodes were provided in the original report, this was indicated by “NA” (not available). Accordingly, these patients were excluded from the respective subgroup analysis. Publications were monitored to eliminate repeatedly reported cases.

**A) Survivors with early diagnosis and normal neurocognitive outcome (n= 39)**

| **Authors** | **Age at diagnosis** | **Neurocognitive outcome** | **Age at onset** | **Number of catabolic episodes prior to diagnosis** | **Number of catabolic episodes following the diagnosis** |
| --- | --- | --- | --- | --- | --- |
| Allen et al. 1969 | neonate | normal | 8 days | NA | NA |
| Lott et al. 1972 | neonate | normal at age 26 months | 12 days | 1 | NA |
| Levy et al. 1973 | neonate 1 | low average IQ 86 (Stanford-Binet Intelligence Scale), IQ 90 (Merrill-Palmer Scale of Mental Tests) at age 2 10/12 years | 10 days | 1 | 1 |
|  | neonate 2 | normal | 1^st^ month | NA | NA |
| Shih et al. 1973 | neonate | normal at age 5 years | NA | NA | NA |
| Saudubray et al. 1976 | neonate 1 | normal at age 9 months | NA | 1 | 1 |
|  | neonate 2 | normal at age 3 months | 2 days | 1 | 0 |
| Mises et al. 1977 | neonate | normal | NA | NA | NA |
| Niederhoff et al. 1977 | neonate | normal, particularly no signs of movement disorder at age 6 months | 30 days | 1 | NA |
| Cohn et al. 1978 | neonate 1 | normal at age 6 months | 3 days | 1 | 0 |
|  | neonate 2 | normal at age 13 months | 12 days | 1 | 0 |
| Lehnert et al. 1979 | neonate | normal | 14 days | 1 | NA |
| Kelleher et al. 1980 | neonate 1 | normal at age 2 years | 3 days | NA | 0 |
|  | neonate 2 | appropriate at age 17 months | 11 days | NA | 0 |
| Bakkeren et al. 1982 | neonate | normal | 6 days | 1 | NA |
| Rousson et al. 1984 | neonate 1 | normal | NA | NA | NA |
|  | neonate 2 | normal | NA | NA | NA |
|  | neonate 3 | normal | NA | NA | NA |
|  | neonate 4 | normal | NA | NA | NA |
| Beauvais et al. 1985 | neonate | normal | 17 days | 1 | 0 |
| Berry et al. 1988 | neonate 1 | DQ 103 (Gesell) at age 9 months | 3 days | 1 | 0 |
|  | neonate 2 | DQ 115 (Mental Development Index), 99 (Psychomotor Development Index) at age 14 months | 1 day | 1 | 0 |
| Heimler et al. 1988 | neonate | DQ 85 (Bayley Scales of Infant Development) at age 12 months, neurologically normal | 7 days | 1 | 0 |
| Naglak et al. 1988 | neonate | normal | Prenatal diagnosis, emesis on day 3 | 1 | NA |
| Pesce et al. 1991 | neonate | normal at age 6 months | 21 days | 1 | NA |
| Shigematsu et al. 1991 | neonate | normal | NA | 1 | NA |
| Dodelson de Kremer et al. 1992 | neonate | normal | 8 days | NA | NA |
| Orban et al. 1994 | neonate | normal at age 9 months | 11 days | 1 | 1 |
| Lorek et al. 1996 | neonate | Griffiths quotient within the normal range at age 12 months, but with some subscore variability | 3 days | 1 | NA |
| Wei et al. 2004 | neonate | normal at age 3 years | 14 days | 2 | NA |
| Hüner et al. 2005 | neonate | normal at age 19 months | 8 days | 1 | NA |
| Dionisi-Vici et al. 2006 | neonate | normal | 1^st^ month | 1 | 6 |
| Gokcay et al. 2006 | neonate | normal | 8 days | 1 | NA |
| Lee et al. 2007 | 15 days | normal | 7 days | NA | NA |
| Wasant et al. 2008 | 10 days | normal | 3 days | NA | NA |
| Castorina et al. 2008 | neonate | normal | 3 days | 1 | NA |
| Castelnovi et al. 2010 | neonate | normal | 7 days | 1 | recurrent |
| Lund and Lund 2011 | neonate 1 | normal | 6 days | 1 | NA |
|  | neonate 2 | normal | 6 days | 1 | NA |

**B) Survivors with early diagnosis and impaired neurocognitive outcome (n= 7)**

| **Authors** | **Age at diagnosis** | **Neurocognitive outcome** | **Age at onset** | **Number of catabolic episodes prior to diagnosis** | **Number of catabolic episodes following the diagnosis** |
| --- | --- | --- | --- | --- | --- |
| Mises et al. 1977 | neonate | retarded | NA | NA | NA |
| Wilson et al. 1984 | neonate | slightly delayed at age 9 months | 6 days | 1 | NA |
| Berry et al. 1988 | neonate 1 | IQ 82 (Wechsler Intelligence Scale for Children-Revised) at age 7 ½ years | 3 days | 1 | 6 |
|  | neonate 2 | IQ 70 (Wechsler Intelligence Scale for Children-Revised) at age 8 ½ years | 13 days | 1 | 0 |
|  | neonate 3 | IQ 49 (Stanford-Binet Intelligence Scale) at age 3 ½ years | 3 days | 1 | 2 |
| Lee et al. 2007 | 22 days | developmental delay | 10 days | NA | NA |
| Castorina et al. 2008 | neonate | disturbed social relationships with his peers, unsatisfactory school performance | 1 day | 1 | NA |

**C) Survivors with early diagnosis and no details on the neurocognitive outcome (n= 8)**

| **Author** | **Age at diagnosis** | **Neurocognitive outcome** | **Age at onset** | **Number of catabolic episodes prior to diagnosis** | **Number of catabolic episodes following the diagnosis** |
| --- | --- | --- | --- | --- | --- |
| Rabier et al. 1992 | neonate 1 | NA | NA | NA | NA |
|  | neonate 2 | NA | NA | NA | NA |
|  | neonate 3 | NA | NA | NA | NA |
| Ito et al. 1995 | neonate 1 | NA | NA | NA | NA |
|  | neonate 2 | NA | NA | NA | NA |
| Itoh et al. 1996 | neonate 1 | NA | NA | NA | NA |
|  | neonate 2 | NA | NA | NA | NA |
| Kasapkara et al. 2011 | neonate | NA | 3 days | 1 | NA |

**D) Deceased patients with neonatal manifestation (n= 27)**

| **Authors** | **Age at diagnosis** | **Outcome^#^** | **Age at onset** | **Number of catabolic episodes prior to diagnosis** | **Number of catabolic episodes following the diagnosis** |
| --- | --- | --- | --- | --- | --- |
| Newman et al. 1967 | neonate | died | 2 days | 1 | NA |
| Sidbury et al. 1967 | neonate 1 | died at day 9 | 2 days | NA | NA |
|  | neonate 2 | died at day 8 | 4 days | NA | NA |
|  | neonate 3 | died | 1 day | NA | NA |
|  | neonate 4 | died at day 13 | 2 days | NA | NA |
| Allen et al. 1969 | neonate 1 | died at 3 weeks | 1-2 weeks | 1 | NA |
|  | neonate 2 | died at 3 weeks | 1-2 weeks | 1 | NA |
| Spirer et al. 1975 | neonate 1 | died at day 6 | 4 days | 1 | NA |
|  | neonate 2 | died at day 7 | 4 days | 1 | NA |
| Saudubray et al. 1976 | neonate | died at day 9 | 2 days | 1 | NA |
| Blaskovics et al. 1978 | neonate | died at day 8 | NA | NA | NA |
| Fischer et al. 1981 | neonate | died at day 13 | 11 days | 1 | NA |
| Truscott et al. 1981 | neonate | died at day 18 | 2 days | 1 | NA |
| Wysocki et al. 1983 | neonate | died at day 8 | 3 days | 1 | NA |
| Rousson et al. 1984 | neonate 1 | died | NA | NA | NA |
|  | neonate 2 | died | NA | NA | NA |
|  | neonate 3 | died | NA | NA | NA |
|  | neonate 4 | died | NA | NA | NA |
| Saudubray et al. 1984 | neonate 1 | died | 2 days | NA | NA |
|  | neonate 2 | died | 1-2 weeks | NA | NA |
|  | neonate 3 | died | 1-2 weeks | NA | NA |
| Naglak et al. 1988 | neonate | died at 2 weeks | NA | NA | NA |
| Shigematsu et al. 1991 | neonate | died of pneumonia after several years of cerebral palsy | NA | NA | NA |
| Tokatli et al. 1998 | neonate 1 | died | 7 days | 1 | NA |
|  | neonate 2 | died at day 6 | 3 days | 1 | NA |
|  | neonate 3 | died at day 5 | 3 days | 1 | NA |
| Gilbert-Barness et al. 1999 | neonate | died at day 19 | 7 days | 1 | NA |

**^#^**Age at death was not reported for each case.

**E) Patients with late diagnosis and normal neurocognitive outcome (n= 28)**

| **Authors** | **Age at diagnosis** | **Neurocognitive outcome** | **Age at onset** | **Number of catabolic episodes prior to diagnosis** | **Number of catabolic episodes following the diagnosis** |
| --- | --- | --- | --- | --- | --- |
| Malan et al. 1977 | 6 years | IQ 127 (New South African Individual Scale) at age 6 years | NA | NA | 0 |
|  | 3 years | IQ 115 (McCarthy Scales of Children's Abilities) at age 3 3/12 years | 6 months | NA | NA |
| Blaskovics et al. 1978 | 6 years | normal | NA | 14 | NA |
| Yudkoff et al. 1978 | 3 ½ years | normal | NA | 3 | NA |
| Duran et al. 1979 | 4 ½ years | IQ 146* | 11 months | 6 | NA |
|  | 2 ½ years | IQ 106* | 15 months | 4 | NA |
| Shigematsu et al. 1982 | 4 10/12 years | IQ 102 at age 4 10/12 years | 18 months | 5 | NA |
| Bakkeren et al. 1982 | 3 ¾ years | normal | NA | NA | NA |
|  | 5 years | normal | NA | NA | NA |
| Rousson et al. 1984 | 4 months (patient 1) | normal | NA | NA | NA |
|  | 4 months (patient 2) | normal | NA | NA | NA |
| Berry et al. 1988 | 4 months | IQ 97 (Wechsler Preschool and Primary Scale of Intelligence) at age 6 4/12 years | NA | 1 | 1 |
| Naglak et al. 1988 | 5 ½ years | normal | 1^st^ month | 3 | 0 |
| Gerdes et al. 1988 | 5 ½ years | normal | NA | 3 | NA |
| Dodelson de Kremer et al. 1992 | late* (patient 1) | normal | NA | NA | NA |
|  | late* (patient 2) | normal | NA | NA | NA |
| van Hove et al. 1994 | 4 8/12 years | normal, healthy since initiation of therapy | NA | NA | NA |
| Mehta et al. 1996 | 5 years | normal | 4 ½ years | 3 | NA |
| Fries et al. 1996 | 7 years | normal | NA | 3 | NA |
| Attia et al. 1996 | 19 months | normal | 14 months | 2 | 1 |
| Tokatli et al. 1998 | 13 months | normal | 13 months | 1 | 3 |
|  | 5 ½ years | normal | 5 months | 5 | 1 |
| Lin et al. 2007 | 2 months | normal | NA | NA | NA |
| Martin-Hernandez et al. 2009 | 4 years (patient 1) | normal | 4 years | “a few” | “a few” |
|  | 4 years (patient 2) | normal | 1 month | 1 | 0 |
| Lund and Lund 2011 | 2 years | normal | 2 years | 1 | NA |
| Pascarella et al. 2011 | 7 8/12 years | normal | NA | NA | NA |
|  | 2 ½ years | normal | NA | NA | 12 |

* Detailed information was not available.

**F) Patients with late diagnosis and impaired neurocognitive outcome (n= 34)**

| **Authors** | **Age at diagnosis** | **Neurocognitive outcome** | **Age at onset** | **Number of catabolic episodes prior to diagnosis** | **Number of catabolic episodes following the diagnosis** |
| --- | --- | --- | --- | --- | --- |
| Budd et al. 1967/ Shih et al. 1973 | 5 years | mild retardation, IQ 72 at age 21 years (Shih et al. 1984) | > 1 month | NA | NA |
|  | 2 ½ years | mild retardation | 4 days | NA | NA |
| Lott et al. 1972 | 10 years | IQ 55* | NA | NA | NA |
| Guibaud et al. 1973 | 3 4/12 years | DQ 35 (Borel-Maisonny Test) at age 4 2/12 years | 4 days | 4 | NA |
| Levy et al. 1973 | 8 years | IQ 53 (Stanford-Binet Intelligence Scale), IQ 63 (Merrill-Palmer Scale of Mental Tests) at age 7 years | NA | several | NA |
| Krieger et al. 1976 | 5 years | IQ 24 (Stanford-Binet Intelligence Scale) at age 5 years, slow development | 14 days | 12 | 4 |
| Lipinski et al. 1977 | 3 4/12 years | IQ 81* | 6 days | 7 | NA |
| Velazquez et al. 1980 | childhood (before age 3 ¼ years)* | mentally retarded | NA | NA | NA |
| Williams et al. 1981 | 3 ½ years | delayed, especially in language areas and fine motor coordination | 3 weeks | NA | NA |
| Duran et al. 1982 | 9 ½ years | severely retarded | NA | 6 | NA |
| Roe et al. 1984 | 3 ¾ years | delayed | 12 months | 4 | 1 |
| Rousson et al. 1984 | late* (patient 1) | retarded | NA | NA | NA |
|  | late* (patient 2) | retarded | NA | NA | NA |
| de Sousa et al. 1986 | 2 ½ years | DQ 81 (Griffiths Mental Development Scale) | 12 days | 3 | NA |
| Berry et al. 1988 | 3 7/12 years | IQ 68 (Wechsler Intelligence Scale for Children-Revised) at age 12 ½ years | 2 ½ years | 4 | 1 |
|  | 3 10/12 years | IQ 67 (Stanford-Binet Intelligence Scale) and IQ 62 (General Cognitive Index) at age 8 years | 15 days | 4 | 5 |
|  | 23 months | DQ 58 (Gesell) at 23 months, IQ 79 (Wechsler Intelligence Scale for Children-Revised) at age 7 10/12 years | 2 months | 6 | 2 |
| Hou et al. 1990 | 3 ½ years | mild psychomotor retardation | NA | NA | NA |
| Mayatepek et al. 1991 | 2 years | delayed before start of therapy; normal speech development and nearly normal motor development after 30 months of carnitine therapy | NA | 5 | 3 (mild) |
| Dodelson de Kremer et al. 1992 | late* | retarded | NA | NA | NA |
| Tokatli et al. 1998 | 4 years | delayed | NA | NA | NA |
| Lee et al. 1998 | 4 years | satisfactory* | 4 years | NA | NA |
| Sanseverino et al. 2000 | 6 months | severe psychomotor retardation | NA | NA | NA |
| Lee et al. 2007 | 3 years | developmental delay | 5 days | NA | NA |
|  | 4 years | developmental delay | 5 days | NA | NA |
|  | 8 years | developmental delay | 10 days | NA | NA |
| Lin et al. 2007 | 3 years | marked disabilities | NA | NA | NA |
|  | 2 years | marked disabilities | NA | NA | NA |
| Qiu et al. 2008 | 2 7/12 years | obvious psychomotor retardation | 3 days | 4 | NA |
| Wasant et al. 2008 | 6 weeks | severe mental retardation | 7 days | NA | NA |
|  | 11 months | severe mental retardation | 10 days | NA | NA |
| Martin-Hernandez et al. 2009 | 6 years | retarded at age 24 years | 12 months | “a few” | “a few” |
|  | 3 years | retarded at age 18 years | 2 weeks | 1 | 0 |
| Lund and Lund 2011 | 3 years | mildly retarded at age 23 years | NA | NA | 2 |

* Detailed information was not available.

**G) Patients with late diagnosis and no details on the neurocognitive outcome (n= 10**)

| **Authors** | **Age at diagnosis** | **Neurocognitive outcome** | **Age at onset** | **Number of catabolic episodes prior to diagnosis** | **Number of catabolic episodes following the diagnosis** |
| --- | --- | --- | --- | --- | --- |
| Ando et al. 1971 | 7 years | NA | NA | NA | NA |
| Salamino et al. 1994 | 9 years | NA | 9 years | 1 | 0 |
| Kahler et al. 1994 | 4 years (patient 1) | NA | 4 weeks | 1 | NA |
|  | 4 years (patient 2) | NA | 3 years | 3 | NA |
|  | 6 years | NA | 6 years | 1 | NA |
| Weinberg et al. 1997 | 8 years | NA | NA | NA | NA |
| Feinstein et al. 2003 | “early childhood”* | NA | NA | NA | NA |
| Sogut et al. 2004 | 19 months | NA | 19 months | NA | NA |
| Kimmoun et al. 2008 | 7 years | NA | NA | NA | 1 |
| Erdem et al. 2010 | 10 years | NA | 2 years | 3 | NA |

* Detailed information was not available.

**H) Deceased patients with late manifestation (n= 2)**

| **Authors** | **Age at diagnosis** | **Outcome^#^** | **Age at onset** | **Number of catabolic episodes prior to diagnosis** | **Number of catabolic episodes following the diagnosis** |
| --- | --- | --- | --- | --- | --- |
| Dodelson de Kremer et al. 1992 | late* | died | NA | NA | NA |
| Tokatli et al. 1998 | 2 ½ years | died | 2 ½ years | NA | NA |

* Detailed information was not available. **^#^**Age at death was not reported.

**Abbreviations:** DQ, developmental quotient; IQ, intelligence quotient

**SUPPLEMENTARY REFERENCES**

Allen DM, Necheles TF, Rieker R, Senior B: **Reversible neonatal pancytopenia due to isovaleric acidemia, abstract.** *Soc Pediat Res* 1969, **May 2-3:**156.

Ando T, Klingberg WG, Ward AN, Rasmussen K, Nyhan WL: **Isovaleric Acidemia Presenting with Altered Metabolism of Glycine** *Pediatr Res* 1971, **5:**478-486.

Attia N, Sakati N, al Ashwal A, al Saif R, Rashed M, Ozand PT: **Isovaleric acidemia appearing as diabetic ketoacidosis.** *J Inherit Metab Dis* 1996, **19:**85-86.

Bakkeren JA, Sengers RC, Ruitenbeek W, Trijbels JM, Houben ML, Van der Zee SP: **[Isovaleric acidemia: identical biochemical picture in 3 patients with variable clinical manifestations].** *Tijdschr Kindergeneeskd* 1982, **50:**153-159.

Beauvais P, Peter MO, Barbier B: **[Neonatal form of isovaleric acidemia. Apropos of a new case].** *Arch Fr Pediatr* 1985, **42:**531-533.

Berry GT, Yudkoff M, Segal S: **Isovaleric acidemia: medical and neurodevelopmental effects of long-term therapy.** *J Pediatr* 1988, **113:**58-64.

Blaskovics ME, Ng WG, Donnell GN: **Prenatal diagnosis and a case report of isovaleric acidaemia.** *J Inherit Metab Dis* 1978, **1:**9-11.

Budd MA, Tanaka K, Holmes LB, Efron ML, Crawford JD, Isselbacher KJ: **Isovaleric acidemia. Clinical features of a new genetic defect of leucine metabolism.** *N Engl J Med* 1967, **277:**321-327.

Castelnovi C, Moseley K, Yano S: **Maternal isovaleric acidemia: observation of distinctive changes in plasma amino acids and carnitine profiles during pregnancy.** *Clin Chim Acta* 2010, **411:**2101-2103.

Castorina M, Rigante D, Antuzzi D, Sciascia Cannizzaro G, Ricci R: **Different outcome in isovaleric acidemia might be related to unsatisfactory diet compliance.** *Scand J Gastroenterol* 2008, **43:**767-768.

Cohn RM, Yudkoff M, Rothman R, Segal S: **Isovaleric acidemia: use of glycine therapy in neonates.** *N Engl J Med* 1978, **299:**996-999.

de Sousa C, Chalmers RA, Stacey TE, Tracey BM, Weaver CM, Bradley D: **The response to L-carnitine and glycine therapy in isovaleric acidaemia.** *Eur J Pediatr* 1986, **144:**451-456.

Dionisi-Vici C, Deodato F, Röschinger W, Rhead W, Wilcken B: **'Classical' organic acidurias, propionic aciduria, methylmalonic aciduria and isovaleric aciduria: long-term outcome and effects of expanded newborn screening using tandem mass spectrometry.** *J Inherit Metab Dis* 2006, **29:**383-389.

Dodelson de Kremer R, Depetris de Boldini C, Paschini de Capra A, Hliba E, Corbella L: **[Phenotypic expression variation of isovaleric acidemia in Argentinian patients. A long term follow-up].** *Medicina (B Aires)* 1992, **52:**131-140.

Duran M, van Sprang FJ, Drewes JG, Bruinvis L, Ketting D, Wadman SK: **Two sisters with isovaleric acidaemia, multiple attacks of ketoacidosis and normal development.** *Eur J Pediatr* 1979, **131:**205-211.

Duran M, Bruinvis L, Ketting D, Wadman SK, van Pelt BC, Batenburg-Plenter AM: **Isovaleric acidaemia presenting with dwarfism, cataract and congenital abnormalities.** *J Inherit Metab Dis* 1982, **5:**125-127.

Erdem E, Cayonu N, Uysalol E, Yildirmak ZY: **Chronic intermittent form of isovaleric acidemia mimicking diabetic ketoacidosis.** *J Pediatr Endocrinol Metab* 2010, **23:**503-505.

Feinstein JA, O'Brien K: **Acute metabolic decompensation in an adult patient with isovaleric acidemia.** *South Med J* 2003, **96:**500-503.

Fischer AQ, Challa VR, Burton BK, McLean WT: **Cerebellar hemorrhage complicating isovaleric acidemia: a case report.** *Neurology* 1981, **31:**746-748.

Fries MH, Rinaldo P, Schmidt-Sommerfeld E, Jurecki E, Packman S: **Isovaleric acidemia: response to a leucine load after three weeks of supplementation with glycine, L-carnitine, and combined glycine-carnitine therapy.** *J Pediatr* 1996, **129:**449-452.

Gerdes AM, Gregersen N, Ludvigsson P, Güttler F: **A Scandinavian case of isovaleric acidaemia.** *Inherit Metab Dis* 1988, **11:**219-220.

Gilbert-Barness E, Barness LA: **Isovaleric acidemia with promyelocytic myeloproliferative syndrome.** *Pediatr Dev Pathol* 1999, **2:**286-291.

Gokcay G, Baykal T, Gokdemir Y, Demirkol M: **Breast feeding in organic acidaemias.** *J Inherit Metab Dis* 2006, **29:**304-310.

Guibaud P, Divry P, Dubois Y, Collombel C, Larbre F: **[A case of isovaleric acidemia].** *Arch Fr Pediatr* 1973, **30:**633-645.

Heimler R, Hennes H, Khayata S, Sasidharan P, Matalon R: **Isovaleric acidaemia in a premature infant: diagnosis and treatment.** *J Inherit Metab Dis* 1988, **11:**313-314.

Hou JW, Wang TR: **Isovaleric acidemia: report of one case.** *Zhonghua Min Guo Xiao Er Ke Yi Xue Hui Za Zhi* 1990, **31:**262-265.

Hüner G, Baykal T, Demir F, Demirkol M: **Breastfeeding experience in inborn errors of metabolism other than phenylketonuria.** *J Inherit Metab Dis* 2005, **28:**457-465.

Ito T, Kidouchi K, Sugiyama N, Kajita M, Chiba T, Niwa T, Wada Y: **Liquid chromatographic-atmospheric pressure chemical ionization mass spectrometric analysis of glycine conjugates and urinary isovalerylglycine in isovaleric acidemia.** *J Chromatogr B Biomed Appl* 1995, **670:**317-322.

Itoh T, Ito T, Ohba S, Sugiyama N, Mizuguchi K, Yamaguchi S, Kidouchi K: **Effect of carnitine administration on glycine metabolism in patients with isovaleric acidemia: significance of acetylcarnitine determination to estimate the proper carnitine dose.** *Tohoku J Exp Med* 1996, **179:**101-109.

Kahler SG, Sherwood WG, Woolf D, Lawless ST, Zaritsky A, Bonham J, Taylor CJ, Clarke JT, Durie P, Leonard JV: **Pancreatitis in patients with organic acidemias.** *J Pediatr* 1994, **124:**239-243.

Kasapkara CS, Ezgu FS, Okur I, Tumer L, Biberoglu G, Hasanoglu A: **N-carbamylglutamate treatment for acute neonatal hyperammonemia in isovaleric acidemia.** *Eur J Pediatr* 2011, **170:**799-801.

Kelleher JF, Jr., Yudkoff M, Hutchinson R, August CS, Cohn RM: **The pancytopenia of isovaleric acidemia.** *Pediatrics* 1980, **65:**1023-1027.

Kimmoun A, Abboud G, Strazeck J, Merten M, Gueant JL, Feillet F: **Acute decompensation of isovaleric acidemia induced by Graves' disease.** *Intensive Care Med* 2008, **34:**2315-2316.

Krieger I, Tanaka K: **Therapeutic effects of glycine in isovaleric acidemia.** *Pediatr Res* 1976, **10:**25-29.

Lee PJ, Harrison EL, Jones MG, Chalmers RA, Leonard JV, Whipp BJ: **Improvement in exercise tolerance in isovaleric acidaemia with L-carnitine therapy.** *J Inherit Metab Dis* 1998, **21:**136-140.

Lee YW, Lee DH, Vockley J, Kim ND, Lee YK, Ki CS: **Different spectrum of mutations of isovaleryl-CoA dehydrogenase (IVD) gene in Korean patients with isovaleric acidemia.** *Mol Genet Metab* 2007, **92:**71-77.

Lehnert W, Schenck W, Niederhoff H: **[Isovaleric acidemia combined with hypertrophic pylorstenosis (author's transl)].** *Klin Padiatr* 1979, **191:**477-482.

Levy HL, Erickson AM, Lott IT, Kurtz DJ: **Isovaleric acidemia: results of family study and dietary treatment.** *Pediatrics* 1973, **52:**83-94.

Lin WD, Wang CH, Lee CC, Lai CC, Tsai Y, Tsai FJ: **Genetic mutation profile of isovaleric acidemia patients in Taiwan.** *Mol Genet Metab* 2007, **90:**134-139.

Lipinski C, Trefz F, Klett M: **Isovalerianazidämie. Zur Differentialdiagnose des azetonämischen Erbrechens mit Somnolenz und Koma.** *Therapiewoche* 1977, **27:**6725-6728.

Lorek AK, Penrice JM, Cady EB, Leonard JV, Wyatt JS, Iles RA, Burns SP, Reynolds EO: **Cerebral energy metabolism in isovaleric acidaemia.** *Arch Dis Child Fetal Neonatal Ed* 1996, **74:**F211-213.

Lott IT, Erickson AM, Levy HL: **Dietary treatment of an infant with isovaleric acidemia.** *Pediatrics* 1972, **49:**616-618.

Lund AB, Lund AM: **[Isovaleric acidaemia--a rare and serious defect in the metabolism of leucine].** *Ugeskr Laeger* 2011, **173:**1121-1123.

Malan C, Neethling AC, Shanley BC, Gompertz D, Bartlett K, Schraader EB: **Isovaleric acidaemia in two South African children.** *S Afr Med J* 1977, **51:**980-983.

Martin-Hernandez E, Lee PJ, Micciche A, Grünewald S, Lachmann RH: **Long-term needs of adult patients with organic acidaemias: outcome and prognostic factors.** *J Inherit Metab Dis* 2009, **32:**523-533.

Mayatepek E, Kurczynski TW, Hoppel CL: **Long-term L-carnitine treatment in isovaleric acidemia.** *Pediatr Neurol* 1991, **7:**137-140.

Mehta KC, Zsolway K, Osterhoudt KC, Krantz I, Henretig FM, Kaplan P: **Lessons from the late diagnosis of isovaleric acidemia in a five-year-old boy.** *J Pediatr* 1996, **129:**309-310.

Mises J, Moussalli F, Plouin P, Saudubray JM: **[An electroencephalographic study of disorders of amino-acid metabolism during the first days of life (author's transl)].** *Rev Electroencephalogr Neurophysiol Clin* 1977, **7:**371-377.

Naglak M, Salvo R, Madsen K, Dembure P, Elsas L: **The treatment of isovaleric acidemia with glycine supplement.** *Pediatr Res* 1988, **24:**9-13.

Newman CG, Wilson BD, Callaghan P, Young L: **Neonatal death associated with isovalericacidaemia.** *Lancet* 1967, **2:**439-442.

Niederhoff H, Lehnert W, Witt I, Richter O, Limberg J: **[Isovaleric acidemia in a small infant].** *Monatsschr Kinderheilkd* 1977, **125:**466-468.

Orban T, Mpofu C, Blackensee D: **Severe CNS bleeding followed by a good clinical outcome in the acute neonatal form of isovaleric acidaemia.** *J Inherit Metab Dis* 1994, **17:**755-756.

Pascarella A, Rosa M, della Casa R, Andria G, Parenti G: **Isovaleric acidemia.** *J Pediatr Endocrinol Metab* 2011, **24:**399.

Pesce F, Cerone R, Caruso U, Romano C: **Acute neonatal isovaleric acidaemia presented without acidosis or ketonuria.** *J Inherit Metab Dis* 1991, **14:**111.

Qiu WJ, Gu XF, Ye J, Han LS, Bai HT, Wang X, Gao XL, Wang Y, Jin J, Zhang HW: **[Clinical and mutational study of a Chinese infant with isovaleric acidemia].** *Zhonghua Er Ke Za Zhi* 2008, **46:**526-530.

Rabier D, Parvy P, Bardet J, Saudubray JM, Kamoun P: **Alloisoleucine in isovaleric acidaemia.** *J Inherit Metab Dis* 1992, **15:**154-155.

Roe CR, Millington DS, Maltby DA, Kahler SG, Bohan TP: **L-carnitine therapy in isovaleric acidemia.** *J Clin Invest* 1984, **74:**2290-2295.

Rousson R, Guibaud P: **Long term outcome of organic acidurias: survey of 105 French cases (1967-1983).** *J Inherit Metab Dis* 1984, **7 Suppl 1:**10-12.

Salamino F, Di Lisa F, Burlina AB, Menabo R, Barbato R, De Tullio R, Siliprandi N: **Involvement of erythrocyte calpain in glycine- and carnitine-treated isovaleric acidemia.** *Pediatr Res* 1994, **36:**182-186.

Sanseverino MT, Wajner M, Giugliani R: **[Application of a clinical and laboratory protocol for the investigation of inborn errors of metabolism among critically ill children].** *J Pediatr (Rio J)* 2000, **76:**375-382.

Saudubray JM, Sorin M, Depondt E, Herouin C, Charpentier C, Pousset JL: **[Isovaleric acidemia. Study and treatment in 3 brothers].** *Arch Fr Pediatr* 1976, **33:**795-808.

Saudubray JM, Ogier H, Charpentier C, Depondt E, Coude FX, Munnich A, Mitchell G, Rey F, Rey J, Frezal J: **Hudson memorial lecture. Neonatal management of organic acidurias. Clinical update.** *J Inherit Metab Dis* 1984, **7 Suppl 1:**2-9.

Shigematsu Y, Sudo M, Momoi T, Inoue Y, Suzuki Y, Kameyama J: **Changing plasma and urinary organic acid levels in a patient with isovaleric acidemia during an attack.** *Pediatr Res* 1982, **16:**771-775.

Shigematsu Y, Kikawa Y, Sudo M, Kanaoka H, Fujioka M, Dan M: **Prenatal diagnosis of isovaleric acidemia by fast atom bombardment and tandem mass spectrometry.** *Clin Chim Acta* 1991, **203:**369-374.

Shih VE, Mandell R, Tanaka K: **Diagnosis of isovaleric acidemia in cultured fibroblasts.** *Clin Chim Acta* 1973, **48:**437-439.

Shih VE, Aubry RH, DeGrande G, Gursky SF, Tanaka K: **Maternal isovaleric acidemia.** *J Pediatr* 1984, **105:**77-78.

Sidbury JB, Jr., Smith EK, Harlan W: **An inborn error of short-chain fatty acid metabolism. The odor-of-sweaty-feet syndrome.** *J Pediatr* 1967, **70:**8-15.

Sogut A, Acun C, Aydin K, Tomac N, Demirel F, Aktuglu C: **Isovaleric acidaemia: cranial CT and MRI findings.** *Pediatr Radiol* 2004, **34:**160-162.

Spirer Z, Swirsky-Fein S, Zakut V, Legum C, Bogair N, Charles R, Gil-Av E: **Acute neonatal isovaleric acidemia. A report of two cases.** *Isr J Med Sci* 1975, **11:**1005-1010.

Tokatli A, Coskun T, Ozalp I: **Isovaleric acidemia. Clinical presentation of 6 cases.** *Turk J Pediatr* 1998, **40:**111-119.

Truscott RJ, Malegan D, McCairns E, Burke D, Hick L, Sims P, Halpern B, Tanaka K, Sweetman L, Nyhan WL, et al: **New metabolites in isovaleric acidemia.** *Clin Chim Acta* 1981, **110:**187-203.

Van Hove JL, Kahler SG, Millington DS, Roe DS, Chace DH, Heales SJ, Roe CR: **Intravenous L-carnitine and acetyl-L-carnitine in medium-chain acyl-coenzyme A dehydrogenase deficiency and isovaleric acidemia.** *Pediatr Res* 1994, **35:**96-101.

Velazquez A, Prieto EC: **Glycine in acute management of isovalericacidaemia.** *Lancet* 1980, **1:**313-314.

Wasant P, Liammongkolkul S, Kuptanon C, Vatanavicharn N, Sathienkijakanchai A, Shinka T: **Organic acid disorders detected by urine organic acid analysis: twelve cases in Thailand over three-year experience.** *Clin Chim Acta* 2008, **392:**63-68.

Wei CC, Lin WD, Tsai FJ, Wu JY, Peng CT, Tsai CH: **Isovaleric acidemia diagnosed promptly by tandem mass spectrometry: report of one case.** *Acta Paediatr Taiwan* 2004, **45:**236-238.

Weinberg GL, Laurito CE, Geldner P, Pygon BH, Burton BK: **Malignant ventricular dysrhythmias in a patient with isovaleric acidemia receiving general and local anesthesia for suction lipectomy.** *J Clin Anesth* 1997, **9:**668-670.

Williams KM, Peden VH, Hillman RE: **Isovalericacidemia appearing as diabetic ketoacidosis.** *Am J Dis Child* 1981, **135:**1068-1069.

Wilson WG, Audenaert SM, Squillaro EJ: **Hyperammonaemia in a preterm infant with isovaleric acidaemia.** *J Inherit Metab Dis* 1984, **7:**71.

Wysocki SJ, French NP, Grauaug A: **Organic aciduria associated with isovaleric acidemia.** *Clin Chem* 1983, **29:**1002-1003.

Yudkoff M, Cohn RM, Puschak R, Rothman R, Segal S: **Glycine therapy in isovaleric acidemia.** *J Pediatr* 1978, **92:**813-817.
