# Supplementary material for: Clinical and neurocognitive outcome in symptomatic isovaleric acidemia
Source: Orphanet J Rare Dis. 2012 Jan 25;7:9. doi: 10.1186/1750-1172-7-9 (PMC3292949; doi:10.1186/1750-1172-7-9)
Supplement: Additional file 2 — Table S2: Clinical chemical and biochemical parameters analyzed in step II participants with symptomatic isovaleric acidemia. [file 1750-1172-7-9-S2.DOC]

**Table S2:** **Clinical chemical and biochemical parameters analyzed in step II participants with symptomatic isovaleric acidemia**. Blood and urine analyses were performed to investigate for primary or secondary organ involvement and nutritional deficiencies.

| **Blood analyses** | Liver | Alanine transaminase, aspartate transaminase, gamma-glutamyl transpeptidase, alkaline phosphatase, coagulation studies (prothrombin time, partial thromboplastin time, fibrinogen) |
| --- | --- | --- |
|  | Renal | Creatinine, uric acid |
|  | Pancreas | Lipase |
|  | Hematology | Complete blood count including differential blood count |
|  | Muscle | Creatine kinase |
|  | Metabolic markers | Lactic acid, ammonia, blood gas analysis, glucose, lipid status |
|  | Inflammatory markers | C-reactive protein, fibrinogen, erythrocyte sedimentation rate |
|  | Nutritional status | Protein electrophoresis, electrolytes, vitamins (folate, vitamin B12), minerals (zinc, copper, iron), amino acids^a^ |
| **Urine analyses** | Renal | Protein, glucose, immunoglobulin G, microalbumin, transferrin, α_1_-microglobulin, N-acetylglucosaminidase |
|  | Metabolic markers | Organic acids including isovalerylglycine^b^ |
| **Functional studies** | Activity of isovaleryl-CoA dehydrogenase (IVD)^c^ | |
|  | Accumulation of isovaleryl (C5)-carnitine in culture medium of EBV-transformed lymphocytes^d^ | |

^a^ Amino acids in blood were determined after protein precipitation with sulfosalicylic acid on a Biotronik LC3000 amino acid analyzer using ion-exchange chromatography followed by post-column derivatization with ninhydrin.

^b^ Quantification of urine isovalerylglycine was performed by gas chromatography with mass selective detection (Hewlett-Packard GCMS System).

^c^ Activity of IVD was analyzed in Epstein-Barr virus (EBV)-transformed immortalized lymphocyte cell lines using the anaerobic electron-transfer flavoprotein reduction assay as described [6].

^d^ Accumulation of C5-carnitine was analyzed in culture medium of transformed lymphocytes according to the method described previously [1].
